# Role of Oxidative and Nitro-Oxidative Damage in Silver Nanoparticles Cytotoxic Effect against Human Pancreatic Ductal Adenocarcinoma Cells

**DOI:** 10.1155/2018/8251961

**Published:** 2018-08-16

**Authors:** Ewelina Barcińska, Justyna Wierzbicka, Agata Zauszkiewicz-Pawlak, Dagmara Jacewicz, Aleksandra Dabrowska, Iwona Inkielewicz-Stepniak

**Affiliations:** ^1^Department of Medical Chemistry, Medical University of Gdansk, Debinki 1, 80-211 Gdansk, Poland; ^2^Department of Histology, Medical University of Gdansk, Debinki 1, 80-211 Gdansk, Poland; ^3^Faculty of Chemistry, University of Gdańsk, Wita Stwosza 63, 80-308 Gdańsk, Poland

## Abstract

Pancreatic ductal adenocarcinoma is one of the most aggressive human malignancies, where the 5-year survival rate is less than 4% worldwide. Successful treatment of pancreatic cancer is a challenge for today's oncology. Several studies showed that increased levels of oxidative stress may cause cancer cells damage and death. Therefore, we hypothesized that oxidative as well as nitro-oxidative stress is one of the mechanisms inducing pancreatic cancer programmed cell death. We decided to use silver nanoparticles (AgNPs) (2.6 and 18 nm) as a key factor triggering the reactive oxygen species (ROS) and reactive nitrogen species (RNS) in pancreatic ductal adenocarcinoma cells (PANC-1). Previously, we have found that AgNPs induced PANC-1 cells death. Furthermore, it is known that AgNPs may induce an accumulation of ROS and alteration of antioxidant systems in different type of tumors, and they are indicated as promising agents for cancer therapy. Then, the aim of our study was to evaluate the implication of oxidative and nitro-oxidative stress in this cytotoxic effect of AgNPs against PANC-1 cells. We determined AgNP-induced increase of ROS level in PANC-1 cells and pancreatic noncancer cell (hTERT-HPNE) for comparison purposes. We found that the increase was lower in noncancer cells. Reduction of mitochondrial membrane potential and changes in the cell cycle were also observed. Additionally, we determined the increase in RNS level: nitric oxide (NO) and nitric dioxide (NO_2_) in PANC-1 cells, together with increase in family of nitric oxide synthases (iNOS, eNOS, and nNOS) at protein and mRNA level. Disturbance of antioxidant enzymes: superoxide dismutase (SOD1, SOD2, and SOD3), glutathione peroxidase (GPX-4) and catalase (CAT) were proved at protein and mRNA level. Moreover, we showed cells ultrastructural changes, characteristic for oxidative damage. Summarizing, oxidative and nitro-oxidative stress and mitochondrial disruption are implicated in AgNPs-mediated death in human pancreatic ductal adenocarcinoma cells.

## 1. Introduction

Pancreatic cancer is a very debilitating and refractory cancer. Although it accounts for only 3% of all cancers worldwide, it is the fourth leading cause of cancer death [1]. The most common type of pancreatic cancer is adenocarcinoma, a type of exocrine pancreatic cancer which is classified as pancreatic ductal adenocarcinoma [2–4]. Due to the fact that the ethology of pancreatic cancer has not been unequivocally described and an effective pancreatic cancer therapy has not been developed, successful diagnosis and treatment of pancreatic cancer are one of the greatest problems of last-day oncology [2, 3]. In recent years, numerous studies have claimed that AgNPs, due to their unique cytotoxic features, size- and shape-depending, antiproliferative, and apoptosis-inducing activity, can be successfully used as antitumor agents [3–5]. Indeed, AgNP-induced cancer cell death by apoptosis, necroptosis, autophagy, and necrosis have been observed [6, 7]. However, the molecular mechanism involved in the cytotoxicity of AgNPs against cancer cells is still underway to clarify [8]. Some studies indicate that nanocytotoxic effect is caused by induction of oxidative and/or nitro-oxidative stress [9, 10]. Overgeneration of ROS and RNS in cells can result in pathological processes through damage to various cellular components, DNA breaks, and impairment of antioxidant potential and cancerogenesis [11]. Accordingly, we hypothesized that generation of oxidative and nitro-oxidative stress using AgNPs could be a new anticancer strategy in the future. During the last decades, it has become clear that ROS and RNS may also play an important role in cell cycle regulation and takes part in stress-induced programmed cells death [12]. Modulation of ROS and RNS metabolism and recruitment of cells to the sensitive phase of the cell cycle can have a positive therapeutic impact in anticancer strategy [13]. ROS are essential secondary messengers in multiple signalling pathways leading to cell death including necrosis, autophagy, mitotic catastrophe, and apoptosis [14, 15]. Oxidative stress-induced programed cells death could be associated with mitochondrial membrane depolarization and mitochondrial remodelling through fission, fusion, or mitophagy [16, 17]. On the other hand, it has been documented that ROS play a crucial role in the transformation of nonmalignant to malignant cells and survival of cancer cells [18–20]. Furthermore, the effects of AgNP-associated metabolic disorders and damage to the antioxidant system has already been demonstrated in cancer cells [21, 22]. Reduction of level as well as activity of superoxide dismutase in cells emerges rapidly as a novel target for cancer therapy [23]. Importantly, it has been noticed that the SOD1 gene is overexpressed in cancers cells [24]. Considering the above-mentioned findings, the aim of our study was to evaluate the cytotoxic effect of AgNP in relation to oxidative and nitro-oxidative stress generation, antioxidant system impairment, mitochondrial damage, and cell cycle disturbance in human pancreatic ductal adenocarcinoma cells.

## 2. Materials and Methods

### 2.1. Chemicals

AgNPs (2.6 nm and 18 nm suspended in water) were purchased from the US Research Nanomaterials, USA.

### 2.2. Characterization of AgNPs

The characteristics of the AgNPs have been presented in our previous study [6, 25, 26]. We showed stability and monodispersity of 2.6 and 18 nm AgNPs in SF culture. We confirmed the negative charge, regular, spherical shape and the presence of silver elements (Ag) in water suspension by TEM with EDS. We described sizes ranging from 1–5 nm with a mean diameter of 2.6 ± 0.8 nm for smaller AgNPs and a size range of 10–26 nm with a mean diameter of 18 ± 2.6 nm for the bigger ones. The released Ag^+^ from 2.6 AgNPs and 18 nm AgNPs was 2.8 *μ*g/mL and 0.66 *μ*g/mL, respectively.

### 2.3. Cells Culture

Human pancreatic ductal carcinoma PANC-1 (CRL-1469) and immortalized human pancreas duct epithelial cell line hTERT-HPNE (CRL-4023) were obtained from the American Type Culture Collection (ATCC). Cells were maintained as a monolayer culture in T-75 cm^2^ tissue culture flasks. PANC-1 cells were cultured in Dulbecco's modified Eagle's medium with high concentration of glucose (ATCC, cat. number: 30-2002), supplemented with 100 *μ*g/mL fetal bovine serum (FBS), 100 *μ*g/mL of penicillin, and 100 *μ*g/mL of streptomycin, and hTERT-HPNE cells were cultured in a mixture of Dulbecco's modified Eagle's medium without glucose (Sigma-Aldrich, cat. number: D-5030) and Medium M3 Base (Incell Corp., cat. number: M300F-500) (3 : 1 ratio) with 2 mM L-glutamine adjusted to 1.5 g/L sodium bicarbonate and supplemented with 5% FBS, 10 ng/mL human recombinant EGF, 5.5 mM D-glucose (1 g/L), and 750 ng/mL puromycin. Cell lines were cultured in standard condition: at 37°C in a humidified atmosphere of 95% O_2_, 5% CO_2_.

### 2.4. Treatments

AgNPs concentrations and exposure time used in these experiments were carefully selected according to our previous study showing the half maximal inhibitory concentration (IC_50_) for PANC-1 cells [6, 25, 26]. To prevent aggregation, AgNPs solutions were shaken for 1 minute before usage (according to the manufacturer's protocol). AgNPs were suspended *ex tempore* in serum free (SF) cell culture medium without FBS and then diluted to the appropriate concentrations.

### 2.5. Determination of ROS Level

Generation of intracellular ROS level was determined by flow cytometry, detected by 2′7′-dichlorofluorescein (H_2_DCF-DA) (Merck, Poland). PANC-1 and hTERT-HPNE cells were seeded into 6-well plates at a density of 2 × 10^4^. The next day, cells were treated with 2.6 or 18 nm AgNPs in concentrations of 0.5, 1.5, 2.5, 3.5, 5 and 5, 10, 25, 50, 100 *μ*g/mL for 24 h as indicated in Treatments. Afterwards, PANC-1 and hTERT-HPNE cells medium were removed, and 10 *μ*M DCF-DA was added to the well for 0.5 h at 37°C. Next, the cells were detached with trypsin solution, washed with PBS, and suspended in 1 mL PBS. Fluorescence of oxidized DCF was measured by flow cytometer at excitation and emission wavelengths of 480 and 525 nm. 10,000 individual cells were measured. The results were expressed as a percent of control.

### 2.6. Determination of NO Level

NO level in PANC-1 cells was determined by the Muse Cell Analyzer, using Muse® Nitric Oxide Kit (Merck, Poland). The cells were seeded into 6-well plates at a density of 2 × 10^4^ cells/well. After 24 h culturing, PANC-1 cells were treated with 2.6 AgNPs in concentrations of 0.5, 1.5, and 2.5 *μ*g/mL and 18 nm AgNPs in concertations of 10, 25, and 50 *μ*g/mL for 24 h as indicated in Treatments. Then, the cells were pelleted and incubated for 30 min with membrane-permeable novel reagent DAX-J2 Orange (Muse Nitric Oxide Reagent, Merck Millipore), according to the manufacturer's protocol. Afterwards, samples cells were analyzed (5000 events/sample) using the Muse Cell Analyzer. The Muse 1.4 analysis software was used to analyze the obtained results.

### 2.7. Assessment of NO_2_ Concentration

Nitrogen dioxide levels in the cytosolic fraction of pancreatic cells (PANC-1 and hTERT-HPNE) were determined on the basis of a standard curve created according to the author's method of Jacewicz [27]. PANC-1 and hTERT-HPNE cells were seeded into 6-well plates at a density of 2 × 10^4^ cells/well for 24 h and next, treated with 2.6 and 18 nm AgNPs in concentrations of 0.5, 1.5, and 2.5 *μ*g/mL and 10, 25, and 50 *μ*g/mL for 24 h as indicated in Treatments. Afterwards, the cells were pelleted and fixed in a 200 mM ice-cold phosphate buffer, pH 7.4. The cytosolic fraction was obtained after 20 min of centrifugation at 100,000 ×g and used for NO_2_ assessment. Samples were prepared by addition of appropriate amounts of the linsidomine (SIN-1) solution and completion of the appropriate buffer to final 3 mL volume. A solution of the complex used to determine NO_2_ concentration was prepared by mixing 0.5 mL of cis-[Cr(C_2_O_4_)(AaraNH_2_)(OH_2_)_2_]^+^ (10^−3^ M) with 2 mL of 0.2 M MES and 2 mL of 2 M NaClO_4_. The temperature was maintained at 20°C with an accuracy of ± 0.1°C. Nitric dioxide concentrations were computed using Origin 6.0 software on the basis of absorbance variations at 541 nm using the nonlinear least squares method [28].

### 2.8. Western Blot Analysis

Protein levels of antioxidant enzymes (SOD1, SOD2, GPX-4, iNOS, eNOS, and nNOS) were detected using Western blotting method. PANC-1 cells were cultured in 10 cm Petri dishes until it reached about 90% confluence. Afterwards, cells were treated with 2.6 and 18 nm AgNPs at concentrations of 0.5, 1, 1.5, 2, 2.5, and 3 *μ*g/mL or 5, 10, 25, and 50 *μ*g/mL and incubated 24 h as indicated in Treatments. Next, culture media was removed; cells were resuspended three times with ice-cold PBS, detached, and centrifuged at 1500 rpm/min for 5 min at room temperature. Then, the supernatant was discharged; cells were homogenized with protein lysis buffer (50 mM Tris pH 7.5, 150 mM NaCl, 1% Triton X-100, 0.1% SDS) in the presence of protease inhibitor (Roche, cat. number: 04693159001). The protein concentrations were measured by the Bradford method [29]. After electrophoresis, proteins were transferred onto nitrocellulose membrane (Protran®, Schleicher and Schuell BioScience) and detected using antibodies: anti-SOD1, anti-SOD2, anti-GPX-4, anti-iNOS, anti-eNOS, and anti-nNOS. *β*-Actin was used as a loading control. The immunoactive proteins were determined using an enhanced chemiluminescence (ECL) Western blotting detection kit (Amersham Biosciences, Piscataway, NJ, USA). Protein levels were quantified using densitometry software (ImageQuant Software).

### 2.9. Real-Time PCR

Changes in genes expression were analyzed by real-time PCR. PANC-1 cells were cultured in 10 cm Petri dishes about 90% confluence. Next, cells were incubated for 24 h with 2.6 and 18 nm AgNPs at concentrations of 0.5, 1.5, and 2.5 *μ*g/mL or 5.10, 25, and 50 *μ*g/mL as indicated in Treatments. After treatment, total RNA was extracted from cell cultures using the ExtractMe Total RNA Plus Kit (Blirt, Poland) according to the manufacturer's instructions. The concentration and purity of isolated RNA were measured with an Epoch spectrophotometer (BioTek, Winooski, USA). Two micrograms of total RNA were subjected to reverse transcription using a RevertAid™ First Strand cDNA Synthesis kit (Thermo Fisher Scientific Inc., USA). The primers used for PCR amplification are listed in Table 1. The reactions were performed in duplicate for each primer set with SensiFAST SYBR No-ROX PCR Master Mix (Bioline, UK). 2 *μ*L of cDNA diluted 5-fold and 200 nM of each primer pair. The PCR conditions were 95°C for 2 min followed by 40 cycles of denaturation for 5 sec at 95°C, annealing for 10 sec at primer-specific temperature, extension for 15 sec at 72°C, and fluorescence reading for 10 sec at 79°C. Dynamic melting curve analysis was performed for all reactions. A total reference RNA (Stratagene) was used to generate a standard curve. The data were collected using the StepOnePlus™ Real-Time PCR System (Life Technologies, USA). The amount of amplified product for each gene was compared to that for the reference gene (RPL37) using a comparative ΔΔCT method and presented as a fold change ± SD.

### 2.10. Cell Cycle

Cell cycle analysis was performed using flow cytometry, detected by propidium iodide (PI) (Merck, Poland). PANC-1 cells were seeded at a density of 2 × 10^4^ into 6-well plates. After 24 h culturing, cells were treated with 2.6 or 18 nm AgNPs in concentrations of 0.5, 1.5, and 2.5 *μ*g/mL and 10, 25, and 50 *μ*g/mL for 24 h as indicated in Treatments. Afterwards, the cells were collected, washed twice with ice-cold PBS, and fixed 24 h at 4°C with 70% ethanol/30% PBS solution. The samples were centrifuged, and the ethanol was removed. Subsequently, cells were resuspended in PBS containing RNAse A (50 *μ*g/mL) and PI (50 *μ*g/ml) and incubated for 0.5 h in room temperature in the dark. PANC-1 cells were analyzed by flow cytometry (BD FACSCalibur™) and CellQuest Pro software. Debris and doublets have been removed by gated appropriate population on FSC/SSC and FL2-A/FL2-W plots before analysis. The percentage of cells in each cell cycle phase was determined by using markers set within the analysis program.

### 2.11. Changes in Mitochondrial Membrane Potential

Changes in mitochondrial membrane potential were analyzed by Muse Cell Analyzer, using the Muse MitoPotential Assay Kit (Merck, Poland). PANC-1 cells were seeded at a density of 2 × 10^4^ per well in 6-well plates. After 24 h of culturing in the standard medium, the cells were treated with 2.6 nm or 18 nm as indicated in Treatments in the concentrations range of 0.5–5 *μ*g/mL or 5–100 *μ*g/mL for 24 h. Then, PANC-1 cells were detached and incubated in 5% CO_2_ at 37°C with the MitoPotential working solution and Muse MitoPotential 7-AAD reagent according to the manufacturer's protocol (Merck, Poland) [30]. The results were analyzed using Muse 1.4 analysis software.

### 2.12. Transmission Electron Microscope

Transmission electron microscope (TEM) analysis was performed at 100 kV (JEM 1200EX II, Jeol, Japan). PANC-1 cells were cultured in 10 cm Petri dishes and treated with 2.6 nm AgNPs in concentrations of 0.5, 2.5, and 5 *μ*g/mL or 18 nm AgNPs in concentrations of 5, 10, 25, 50, and 100 *μ*g/mL as indicated in Treatments. Afterwards, cells were fixed in 2.5% glutaraldehyde in 0.1 mM sodium-cacodylate buffer and prepared according to the previously described method [6, 25].

### 2.13. Statistical Analysis

The obtained data were expressed as mean ± SD for triplicate determination of 3-4 separate experiments. The results were analyzed using one-way ANOVA and Tukey's post hoc test, and *p* value < 0.05 was considered statistically significant.

## 3. Results

### 3.1. Increase in ROS Level in PANC-1 Cells after Treatment with AgNPs

**Figure 1:**
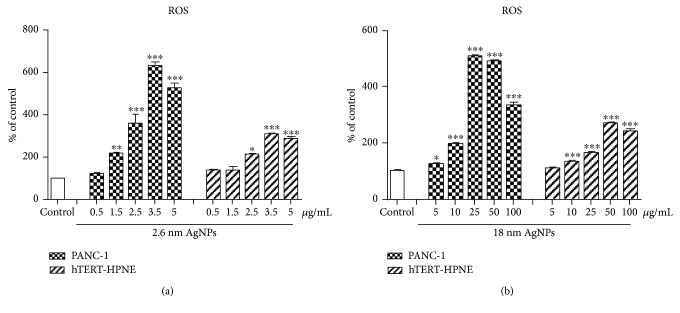
Increase in ROS level in PANC-1 and hTERT-HPNE cells after treatment with 2.6 nm and 18 nm AgNPs for 24 h. Data are mean ± SD of 3 separate determinations. ^∗^*p* < 0.05; ^∗∗^*p* < 0.01; ^∗∗∗^*p* < 0.001 treatments versus control.

We investigated the endogenous ROS level in pancreatic cancer cells after 24 h exposure to AgNPs compared with nontumor cells of the same tissue, and we found increased ROS level in both cell lines (see Figure 1). However, ROS level was about 2 times lower in hTERT-HPNE cells than in PANC-1 cells after treatment with 2.6 nm AgNPs at concentrations of 1.5, 2.5, 3.5, and 5 *μ*g/mL or 18 nm AgNPs at concentrations of 10, 25, 50, and 100 *μ*g/mL. Moreover, incubation of pancreatic cancer cells with 2.6 nm AgNPs (at a concentration of 3.5 *μ*g/mL) led to a 6-fold increase in intracellular ROS level as compared with control cells. 18 nm AgNPs at concentrations of 25 and 50 *μ*g/mL accelerated ROS production to 4-fold above control values. On the other hand, we noticed a slight decrease of ROS level in PANC-1 cells treated with highest concentrations (5 *μ*g/mL and 100 *μ*g/mL) of 2.6 and 18 nm AgNPs.

Next, we investigated the role of RNS: NO and NO_2_ in AgNP-induced pancreatic cancer cells death.

### 3.2. Impact on RNS: NO and NO_2_ and Nitric Oxide Synthases Protein and mRNA Level in PANC-1 Cells after Treatment with AgNPs

**Figure 2:**
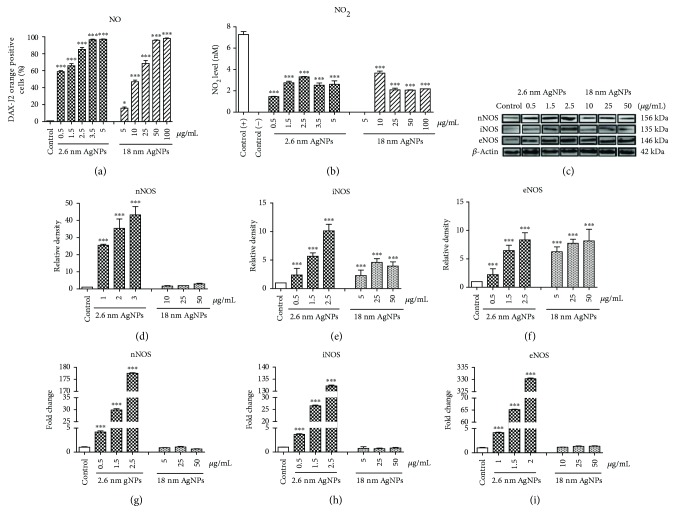
2.6 and 18 nm AgNPs induced RNS generation and changes in NOS protein and mRNA level in PANC-1 cells. (a) NO. (b) NO_2_, changes in NOS protein level: (c) representative Western blot analysis of nNOS, iNOS, and eNOS. (d) nNOS, (e) iNOS, and (f) eNOS. mRNA level of (g) nNOS, (h) iNOS, and (i) eNOS in PANC-1 cells after treatment for 24. Control (+): cells treated with LPS for 24 h; control: untreated cells. Data are expressed as mean ± SD of 4 independent experiments. ^∗^*p* < 0.05; ^∗∗∗^*p* < 0.001 exposed cells versus control.

We demonstrated that 24 h incubation of PANC-1 cells with 0.5–5 *μ*g/mL of 2.6 nm AgNPs or 5–100 *μ*g/mL of 18 nm AgNPs caused an increase of NO level in a concentration-dependent manner (see Figure 2(a)). The level of NO was 58.8% in cells incubated with minimal concentration (0.5 *μ*g/mL) and about 97% in cells incubated with highest (3.5 and 5 *μ*g/mL) concentration of 2.6 nm AgNPs when untreated cells showed 1% of NO. In PANC-1 cells treated with 5, 10, 25, 50, and 100 *μ*g/mL, 18 nm AgNPs level of NO was significantly augmented by 15.8%, 45.7%, 65.3%, 95.8%, and 98.1%, respectively. An increase of NO level after 24 h treatment with 5 *μ*g/mL of 2.6 nm AgNPs was 6-fold higher than after incubation with the same concentration of 18 nm AgNPs. Moreover, we demonstrated 2.6 nm and 18 nm AgNP-induced generation of NO_2_ in PANC-1 cells (see Figure 2(b)). This effect was not dependent on the concentration of AgNPs. For all used concentrations of 2.6 and 18 nm AgNPs, the NO_2_ level was maintained at 2-3 nM, while positive control (treated with LPS) showed NO_2_ level about 7 nM.

Next, we noticed that 2.6 nm AgNPs induced a significant increase in iNOS, nNOS, and eNOS protein level in PANC-1 cells and 18 nm AgNPs induced a significant increase in eNOS and iNOS protein level (see Figures 2(d)–2(f)). Interestingly, the highest increase we observed in nNOS level (50-fold higher than control) for 3.5 *μ*g/mL 2.6 nm AgNPs and in eNOS protein level for 2.5 *μ*g/mL 18 nm AgNPs (5-fold higher than control).

Furthermore, our result showed that 2.6 nm AgNPs induced increasing of all NOS isoforms (iNOS, eNOS, and nNOS) at mRNA level in a concentration-dependent manner (see Figures 2(f)–2(h)). The highest increase we observed for eNOS, which was 2-fold higher as the other isoforms. On the other hand, bigger AgNPs (18 nm) did not affect the level of investigated isoforms of NOS mRNA (see Figures 2(g)–2(i)).

Additionally, we focused on the determination of protein and mRNA level of selected cellular antioxidant in PANC-1 cells after treatment with AgNPs.

### 3.3. Impairment of Enzymatic Antioxidant Defense System in PANC-1 Cells after Treatment with AgNPs

**Figure 3:**
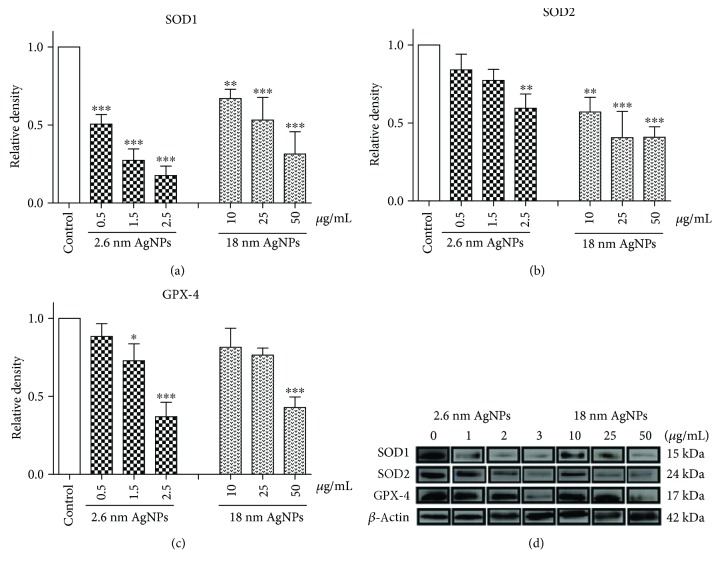
2.6 and 18 nm AgNPs induced a decrease in antioxidant enzymes protein level in PANC-1 cells after treatment for 24 h. (a) SOD1. (b) SOD2. (c) GPX-4. (d) Representative Western blot analysis of SOD1, SOD2, and GPX-4. *β*-actin was used as internal control. Data are mean ± SD of 3 separate determinations. ^∗^*p* < 0.05; ^∗∗^*p* < 0.01; ^∗∗∗^*p* < 0.001 as compared with untreated cells.

**Figure 4:**
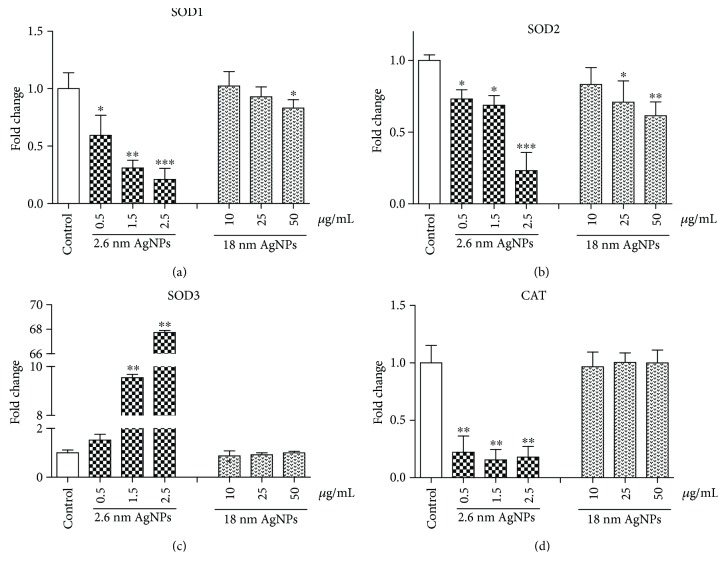
2.6 and 18 nm AgNP-induced changes in antioxidant enzymes at mRNA level in PANC-1 cells after treatment for 24 h. (a) SOD1. (b) SOD2. (c) SOD3. (d) CAT. Data are expressed as mean ± SD of 4 independent experiments. ^∗^*p* < 0.05; ^∗∗^*p* < 0.01; ^∗∗∗^*p* < 0.001 exposed cells versus control.

We evaluated the protein level of selected antioxidant enzymes: SOD1, SOD2, and GPX-4. We observed a significant reduction in cytosolic and mitochondrial SOD and GPX-4 at protein level (see Figure 3). We noticed a statistically significant decrease of SOD1 after treatment with both 2.6 and 18 nm AgNPs in the entire range of used concentrations. Interestingly, bigger AgNPs induced a higher decrease than 2.6 nm AgNPs in SOD2 protein level, while smaller ones caused a higher decrease of SOD1 protein level. In a parallel study, we showed that 2.6 nm AgNPs caused a higher decrease in SOD1, SOD2, and CAT at mRNA level after 24 h incubation than 18 nm AgNPs (see Figure 4). Moreover, after treatment PANC-1 cells with 2.6 nm AgNPs, we observed a lower reduction in SOD2 mRNA level than in SOD1 mRNA level, contrary to the protein level. We have not noticed a significant change in CAT mRNA level after treatment with 18 nm AgNPs. On the other hand, we found a significant increase in SOD3 mRNA level after treatment with 2.6 nm AgNPs in a concentration-dependent manner, while bigger AgNPs did not affect the expression of this SOD isoform.

Subsequently, we decided to explore more deeply the implication of oxidative and nitro-oxidative stress in AgNPs-induced pancreatic cancer cell death.

Thus, next, we have estimated the effect of AgNPs with both sizes on cell cycle in PANC-1.

### 3.4. Cell Cycle Distribution after Treatment PANC-1 Cells with AgNPs

**Figure 5:**
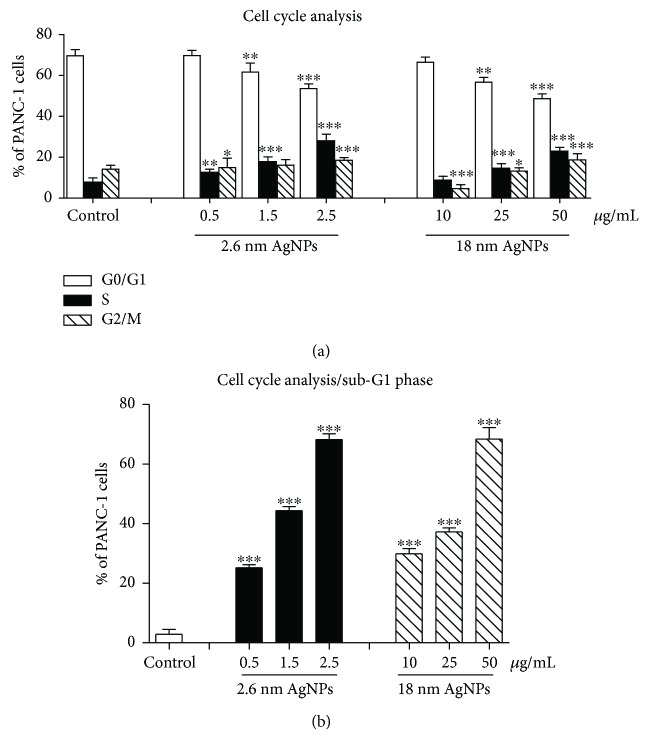
The cell cycle distribution of PANC-1 cells treated with 2.6 nm and 18 nm AgNPs for 24 h incubation. (a) The percentage of cells in each phase (G0/G1, S, and G2/M). (b) Cell population in a sub-G1 fraction. Results are given as mean ± SD of 3 separate determinations. ^∗^*p* < 0.05; ^∗∗^*p* < 0.01; ^∗∗∗^*p* < 0.001 as compared with untreated cells.

After 24 h incubation with 2.6 nm and 18 nm AgNPs, we noticed a decrease of G0/G1 phase cell population in a concentration-dependent manner compared with control (Figure 5(a)). 24 h incubation with 0.5, 1.5, and 2.5 *μ*g/mL of 2.6 nm and 25 and 50 *μ*g/mL of 18 nm AgNPs resulted in a significantly higher percentage of cells in the S phase (12.9 ± 1.5%, 17.7 ± 2.1%, 28.6 ± 2.3%, 4.6 ± 1.6%, and 23.3 ± 2.7%, resp.) than in control (7.9 ± 0.8%). Furthermore, we noticed an increase of G2/M phase for cells treated with both 2.6 nm and 18 nm AgNPs at all used concentrations. Importantly, as shown in Figure 5(b) a significant increase in the percentage of cells in the sub-G1 phase was found after treatment with 2.6 nm AgNPs at 0.5, 1.5, and 2.5 *μ*g/mL (25.3 ± 0.8%, 44.3 ± 1.8%, and 66 ± 1.2%, resp.) and 18 nm AgNPs at 10, 25, and 50 *μ*g/mL (29.2 ± 1.6%, 37.3 ± 2.4, and 68.2 ± 2.1%, resp.) in comparison with control (1.8 ± 1.10%).

Importantly, as shown in Figure 5(b) a significant increase in the percentage of cells in the sub-G1 phase was found after treatment with 2.6 nm AgNPs at 0.5, 1.5, and 2.5 *μ*g/mL (25.3 ± 0.8%, 44.3 ± 18%, and 66 ± 1.2%, resp.) and 18 nm AgNPs at 10, 25, and 50 *μ*g/mL (29.2 ± 1.6%, 37.3 ± 2.4, and 68.2 ± 2.1%, resp.) in comparison with control (1.8 ± 1.10%).

Afterwards, we decided to examine the effect of AgNPs on the mitochondrial membrane potential and ultrastructure of mitochondria in PANC-1 cells.

### 3.5. Changes in Mitochondrial Membrane Depolarization and Ultrastructural Alterations in PANC-1 after Treatment with AgNPs

**Figure 6:**
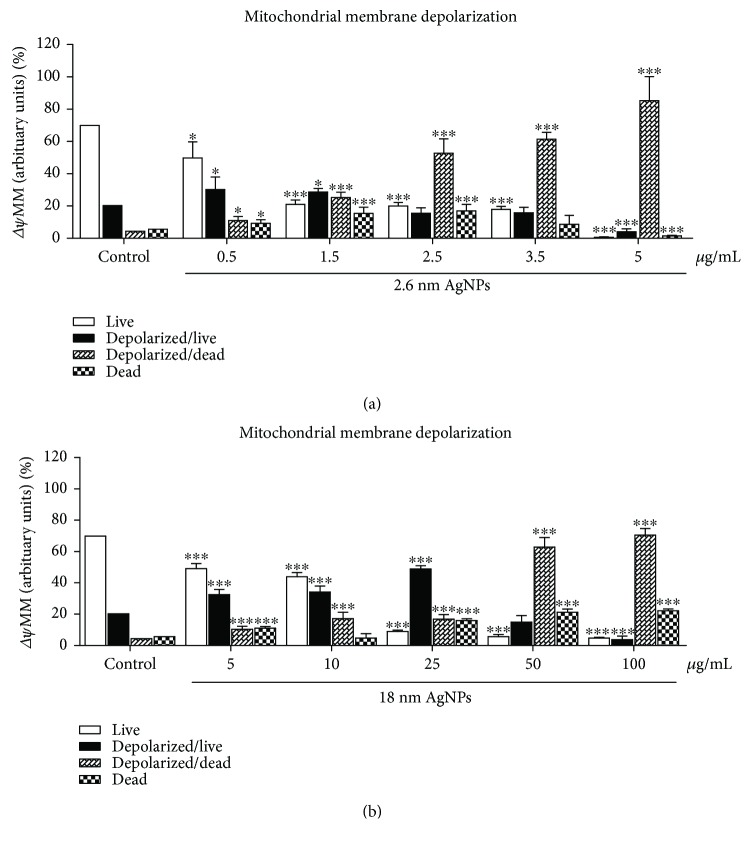
(a) 2.6 nm and (b) 18 nm AgNPs induced changes in mitochondrial membrane potential of PANC-1 cells after 24 h treatment. Changes in mitochondrial membrane potential and induction of cell death were determined using the Muse Cell Analyzer. Values are the mean ± SD of 3 independent experiments. ^∗^*p* < 0.05; ^∗∗∗^*p* < 0.001 exposed cells versus untreated control.

We found that 24 h treatment with 2.6 and 18 nm AgNPs resulted in an increase of the percentage of cells with low mitochondrial membrane potential (Δ*ψ*m), compared to the untreated cells (see Figure 6) in a concentration-dependent manner.

**Figure 7:**
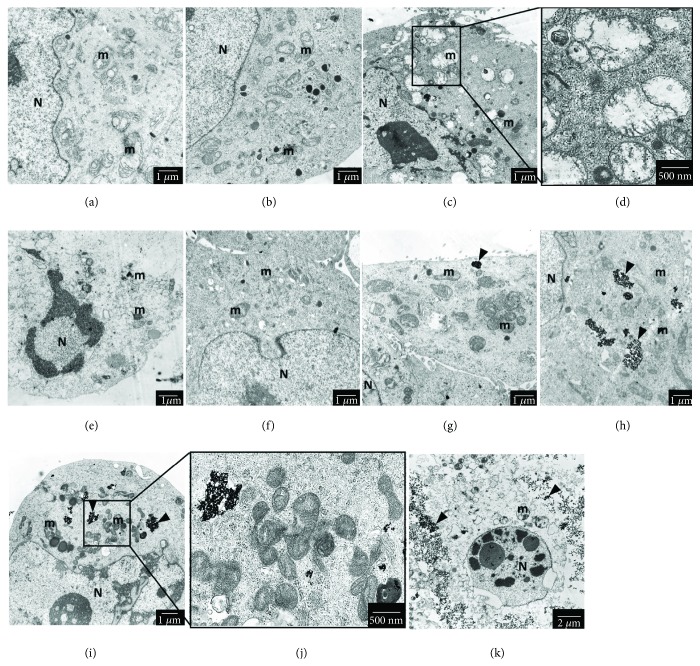
The ultrastructure of mitochondria in PANC-1 cells after treatment with 2.6 nm and 18 nm AgNPs for 24 h. (a) untreated PANC-1 cells. (b, c/d, e) cells treated with 2.6 nm AgNPs at concentrations of 0.5, 2.5, and 5 *μ*g/mL, respectively. (f, g, h, i/j, k) cells treated with 18 nm AgNPs at concentrations of 5, 10, 25, 50, and 100 *μ*g/mL, respectively. N: nucleus; m: mitochondria; arrow: AgNPs. Magnifications: (a–h) ×6000; (i) ×5000; (k) ×4000. Scale bars: (a–c) and (e–i) 1 *μ*m; (k) 2 *μ*m; (d, j) 500 nm.

This effect was accompanied by alteration of mitochondrial ultrastructure (see Figure 7). Unaltered mitochondrial ultrastructure with long, regular cristae, electron-lucent matrix, and oval-to-rod-shape were observed in a control sample (see Figure 7(a)), cells treated with 0.5 *μ*g/mL of 2.6 nm AgNPs (see Figure 7(b)) and cells treated with 5, 10, and 25 *μ*g/mL of 18 nm AgNPs (see Figures 7(f)–7(h)). We noticed swelling of mitochondria and degradation of cristae in cells treated with 2.6 nm AgNPs in a concentration of 2.5 *μ*g/mL (see Figures 7(c) and 7(d)). In higher concentration of 5 *μ*g/mL, we observed general cell degradation and nuclear condensation leading to cell death (see Figure 7(e)). Also, we observed shrinkage and condensation of mitochondria in the presence of 18 nm AgNPs in a concentration of 50 *μ*g/mL (see Figures 7(i) and 7(j)). In the highest concentration of 18 nm AgNPs (100 *μ*g/mL), we observed cell death, accompanied by nuclear condensation and cell organelles degradation, such morphology suggested cell death (see Figures 7(k) and 7(j)).

## 4. Discussion

To assess the role of oxidative stress in AgNP-induced pancreatic cancer cell death, we designated AgNP-concentration range based on our earlier study [6] and the half maximal inhibitory concentration (IC_50_) value determination, which is presented in Table 2.

Importantly, we showed a significantly higher cytotoxic effect of AgNPs on PANC-1 than nontransformed pancreatic cell. In our previous study, we have observed that 2.6 and 18 nm AgNPs induced mixed types of pancreatic cancer cells death [6]. In the present work, we decided to evaluate the contribution of oxidative and nitro-oxidative stress in AgNP-induced cytotoxicity against human pancreatic adenocarcinoma cells because of their important role in cancer cell death. We found that treatment of PANC-1 cells with AgNPs resulted in the enhancement of ROS production. Moreover, this increase was more significant in cancer cells than in noncancer cells of the same tissue. Similarly, Gurunathan et al. [31] documented ROS-mediated mechanism of the mitochondrial pathway of apoptosis after treatment with AgNPs in human breast cancer cells. Also, Vasanth et al. [32] noticed that AgNPs have a great anticancer potential due to selective disruption of the mitochondrial respiratory chain, which leads to the production of ROS, oxidative damage, and ultimately cell death. Edderkaoui et al. [33] showed that ROS production through activation of NADPH oxidase resulted in increased pancreatic cancer cell survival.

Moreover, we reported a significant increase of NO and NO_2_ level in PANC-1 cells after treatment with 2.6 and 18 nm AgNPs. Effective anticancer activity associated with increasing NO level was noticed in pancreatic, colon, and ovarian cancer cells [34–36]. Furthermore, aberrant generation or metabolism of NO increases nitro-oxidative stress and upregulation of the cell death mediators p53 [37]. Indeed, in our previous study, we described a significant increase in the level of p53 protein in PANC-1 cells after treatment with 2.6 and 18 nm AgNPs [6]. We also observed an increase of all investigated NOS isoforms: iNOS, eNOS, and nNOS at mRNA and protein level after 24 h treatment with 2.6 nm of AgNPs and in protein level after 24 h treatment with 18 nm AgNPs. Thus, our data suggested that 2.6 and 18 nm AgNPs regulated NOS protein levels by different pathways: 2.6 nm AgNPs regulates at the transcriptional level, whereas 18 nm AgNPs regulates at the translational level. Similarly, Niu et al. [38] described 1–10 nm CeO2-induced increase in iNOS mRNA level in human cardiomyocytes, and González et al. [39] showed that 45 nm AgNPs induced increase in iNOS protein level in the rat tracheal smooth muscle. Moreover, Xue et al. [40] compared the induction of protein and mRNA iNOS level depending on the type and size of NP in primary microglia cells. They showed the highest iNOS mRNA and protein level in primary microglia cells after 24 h incubation with 20 nm TiO_2_NPs in comparison to 24 h incubation with 12 nm HAPNPs and 20 nm SiO_2_NPs. 20 nm TiO_2_NPs showed a higher increase in protein than in mRNA iNOS level. On the other hand, they noticed a higher mRNA than protein iNOS level after treatment with 12 nm HAPNPs and 20 nm SiO_2_NPs [40]. On the other hand, under *in vitro* exposure, some types of NPs (2.4 nm PtNPs, 3–5 nm CeO_2_, 13 nm AuNPs) have not significantly affected NOS mRNA nor protein level [41–43].

It has been documented that NO and other RNS can be synthesized by two NOS isoforms: eNOS and iNOS in human pancreatic cancer cells [44–46]. However, current research also demonstrated the significant role of nNOS increased overexpression in tumor (lung and ovarian) progression [47, 48]. We present, to the best of our knowledge for the first time, a significant increase of nNOS protein and mRNA level in human pancreatic cancer cells. Our findings are similar to those of Begum et al. [48], who also noticed that an increase in nNOS and iNOS expression lead to RNS production, nitro-oxidative stress, and human lung cancer cells death. Chakraborty et al. [49] observation proved a significant increase of iNOS protein level and NO level in murine fibrosarcoma cells after treatment with 10 nm AgNPs. Xie et al. [50] showed that overexpression of iNOS mRNA level reduced the survival and metastatic potential of a murine melanoma cell line. Also, Jadeski et al. [51] described that iNOS expression in human pancreatic cancer cells is positively correlated with apoptosis. On the other hand, an increase in iNOS expression can affect the stimulation of angiogenesis and tumor progression [52]. However, Kong et al. [44] have not observed a significant role of iNOS in pancreatic cancer cells proliferation and regulation of angiogenesis, which can play a crucial role in tumor growth and metastasis. Interestingly, Díte et al. [46] suggested that eNOS have limited role in the normal physiology of pancreatic cells, while aberration in eNOS expression may play a significant role in cancer cells progression and programmed cell death [22, 46]. On the other hand, the antitumor effects of eNOS inhibition in pancreatic cancer cells were observed [45]. Gratton et al. [53] showed that inhibition of eNOS activity decreases tumor vascular permeability and tumor growth in hepatocarcinoma and lung carcinoma xenograft models.

Furthermore, we demonstrated that AgNP-induced generation of ROS and RNS in PANC-1 cells was associated with a significant disturbance of antioxidant enzymes at protein and mRNA level. It has been indicated that ROS and/or RNS production and antioxidant system impairment lead to programmed cancer cell death [21, 54]. Ahamed et al. [55] reported that ZnONPs significantly decreased the antioxidant level in hepatoma cells and triggered apoptosis. Arora et al. [56] and Jin et al. [57] showed a reduced level of SOD after treatment of human skin carcinoma and human fibrosarcoma with 7–20 nm AgNPs leading to apoptosis. Moreover, Asadpour et al. [58] noticed that < 100 nm ZrO_2_NPs-induced decrease in GPX activity in rat pheochromocytoma, and mouse neuroblastoma cells were related to genotoxic and cytotoxic effect. In PANC-1 cells, we noticed a decreased level of SOD1 protein and mRNA after treatment with 2.6 nm and 18 nm AgNPs. Papa et al. [24] found that inhibition of SOD1 activity in breast cancer cells (MCF-7) leads to a drastic alteration in the morphology of the mitochondria associated with increased fragmentation and swelling of the matrix. This effect was not observed in the nonmalignant breast epithelial cells line (MCF-10A). Glasauer et al. [59] described that SOD1 inhibition drastically reduces lung carcinoma cells proliferation. Moreover, Peng et al. [60] suggested that the mechanism by which SOD1 inhibitors cause cell death is likely through a combination of the regulated mechanism (apoptosis) and unregulated mechanism (oxidative damage to the organelles). In addition, we identified 2.6 and 18 nm AgNPs induced a decrease in SOD2 protein and mRNA level. It has been known that in the mitochondrial matrix, the manganese dismutase SOD2 acts as a major antioxidant enzyme. Mn-SOD low activity and expression have also been reported in certain colorectal carcinomas and pancreatic cancer cells [61]. Ahamed et al. [55] noticed a decrease of SOD1 and SOD2 protein level and activity in human alveolar adenocarcinoma after treatment with 28–38 nm Zn-doped TiO_2_NPs. Moreover, we showed that treatment of PANC-1 cells with 2.6 nm AgNPs resulted in an increase of SOD3 mRNA level, which catalyzes the dismutation of superoxide in the extracellular environment [35]. SOD3 is highly cell-type specific and can be found most abundantly in the pancreas, lung, kidney, and vasculature [34–36]. It has been known that loss of SOD3 level could contribute to aggressive and refractory nature of pancreatic ductal adenocarcinoma. Hayano et al. [62] proved that overexpression of EcSOD in PDA cell lines resulted in decreased invasiveness, slower growth, and peritoneal metastasis of pancreatic cancer cells.

Furthermore, we noticed decreased mRNA level of CAT in PANC-1 cells after treatment with smaller AgNPs. Pramanik et al. [63] showed that overexpression of CAT by transient transfection protected the human squamous carcinoma cells from capsaicin-mediated ROS generation and apoptosis. On the other hand, Glorieux et al. [64] showed that CAT overexpression leads to a less aggressive phenotype and an altered response to chemotherapy of breast cancer cells.

In addition, in PANC-1 cells, we observed a significant reduction of GPX-4 protein level after treatment with 2.6 and 18 nm AgNPs. GPX-4 is considered to be the primary enzymatic defense mechanism against oxidative damage to cellular membranes [65]. Yang et al. [66] reported that GPX-4 is an essential regulator of programmed cancer cell death (ferroptotic). Besides, Hangauer et al. [67] suggested that silencing of GPX-4 may turn out a new strategy to prevent acquired drug resistance. This is related to the demonstrated high resistance of melanoma cells (A375) in a high mesenchymal therapy-resistant to GPX-4 dependence. Loss of GPX-4 function results in selective ferroptotic cell death and prevents tumor relapse in mice.

Summarizing, Glasauer et al. [59] suggested that the reduction of antioxidant enzymes (SOD1, SOD2, GPX-4, and CAT) level induced apoptosis by the activation of p38 and a decrease in the antiapoptotic factor MCL1 caused by elevation in ROS level in lung cancer cells.

Furthermore, we observed that AgNPs caused an increase in PANC-1 cells population with low mitochondrial membrane potential (Δ*ψ*m) in a concentration-dependent manner. Teodoro et al. [68] noticed a size-dependent effect of 40 and 80 nm AgNPs on the activation of the mitochondrial permeability transition. Interestingly, a stronger effect was demonstrated for smaller (40 nm) AgNPs. Moreover, it has been proved that the disruption of mitochondrial membrane potential (Δ*ψ*m) and opening mitochondrial membrane pores are associated with apoptosis or other forms of programmed cell death [69, 70]. Kim et al. [71] indicated that the mitochondrial membrane permeabilization leads to the release of apoptogenic factors and next to apoptosis of prostate cancer cells. Jung et al. [72] described the model of apoptotic pancreatic cancer cells death inducing by mitochondrial membrane depolarization and caspase activation. Besides, some reports showed the harmful effect of nanostructures on mitochondrial function in the lung and kidney cancer cells [73]. These results suggest that AgNP-induced mitochondrial-mediated PANC-1 cell death could occur on the path to apoptosis. Furthermore, some studies described a close link between NP-induced ROS elevation, cell cycle arrested, and mitochondrial-mediated apoptosis [23, 74]. In our study, we noticed a significant increase in sub-G1 population induced by AgNPs, and this observation could be related to PANC-1 apoptotic cell death, which we documented previously [26]. A relation between an increase of sub-G1 hypodiploid cells population and mitochondrial-mediated apoptosis has been described [75, 76]. Similarly, Zhu et al. [75] described cell cycle arrested in sub-G1 phase in human liver cancer cells treated with 2 nm AgNPs. Furthermore, 2.6 nm AgNP-induced increase of S phase in PANC-1 cells could also trigger apoptosis [77]. Salehi et al. [78] reported that chitosan, a semisynthetic biobased polysaccharide, promotes S phase cell cycle arrest and ROS-mediated apoptosis in triple-negative breast cancer cells. On the other hand, Hu et al. [79] described a significant decrease of S phase and apoptosis pathway in breast cancer cells after treatment with an alcohol extract of *Ganoderma lucidum*. Interestingly, Panzarini et al. [80] suggested that AgNPs may be exploited for the development of novel antiproliferative treatment in cancer therapy because of decreasing Hela cell viability, arresting the cell cycle in S, G2/M phase, and increasing sub-G1 population. Zhang et al. [76] reported that AgNP-induced human nasopharyngeal carcinoma and liver cancer cells arrest at G2/M or G1 cell cycle phase resulted in increased radiation sensitivity of tumors. In our study, we noticed that AgNPs treatment leads to cell cycle arrest in S, G2/M-phase in PANC-1 cells. However, Li et al. [81] reported that AgNPs inhibited cell cycle in the G2/M phase in glioblastoma U251 cells, and it could be associated with repair of cell oxidative damage.

Our previous work showed cellular uptake of 2.6 and 18 nm AgNPs in PANC-1 cells and their localization in the cytosol [6]. During this study, we found that AgNP-induced changes in biochemical parameters were associated with ultrastructural alteration of pancreatic cancer cells link to oxidative stress, such as swelling of mitochondria, degradation of cristae, shrinkage, and condensation of mitochondria as well as nuclear condensation [60, 82–84]. These changes have been also described as characteristic of apoptosis via oxidative stress in *in vitro* models [84, 85] and are consistent with our previous study [26].

Considering all the information above, our results indicated that induction of ROS and RNS generation along with disturbance in the enzymatic antioxidant system is implicated in the cytotoxicity activity of AgNPs against pancreatic cancer cells.

## 5. Conclusions

To summarize, we observed that exposure to both 2.6 nm and 18 nm AgNPs leads to an increased production of ROS and RNS in PANC-1 cells. We found upregulation of mRNA and protein level of NOS family, and for the first time, we have described a significant increase of nNOS in pancreatic cancer cell death. This effect was associated with arrested PANC-1 cells in sub-G1cell cycle phase related to programmed cell death, low level of mitochondrial membrane potential, and changes in mitochondrial ultrastructure, typical for oxidative damage. Moreover, we detected AgNP-induced disturbance of antioxidant system (SOD1, SOD2, GPX-4, CAT, and SOD3) in pancreatic cancer cells. In conclusion, we confirmed our hypothesis that oxidative and nitro-oxidative mechanism is implicated in AgNP-induced human pancreatic ductal adenocarcinoma cell death. Our results can be used in developing new strategies for pancreatic cancer nanotherapy.

## Figures and Tables

**Table 1:** The list of primers used in the study.

Gene name	Forward primer	Reverse primer	Annealing temperature
RPL37	TTCTGATGGCGGACTTTACC	CACTTGCTCTTTCTGTGGCA	60
nNOS	CTCACCCCCTCCTTCGAATACC	AAGCTTGCGATTTGCCTGTCTC	60
iNOS	ACGGCTCCTTCAAAGAGGCAAA	TAACGCACGTGTCTGCAGATGT	60
eNOS	ACATGCTGCTGGAAATTGGG	TGGTCCACGATGGTGACTTT	60
bNOS	AAAGCCCACATGGAAAGGCT	AGGTTCCCTTTGTTGGTGGCAT	60
CAT	ACGGGGCCCTACTGTAATAA	AGATGCAGCACTGGAAGGAG	60
SOD1	CCACACCTTCACTGGTCCAT	CTAGCGAGTTATGGCGACG	62
SOD2	TAGGGCTGAGGTTTGTCCAG	CACCGAGGAGAAGTACCAGG	62
SOD3	CGAGTCAGAGTTGGGCTCC	TCTCTTGGAGGAGCTGGAAA	62

**Table 2:** IC_50_ values obtained after treatment of PANC-1 and hTERT-HPNE cells with 2.6 and 18 nm AgNPs.

	IC_50_ from LDH	IC_50_ from MTT	IC_50_ from LDH	IC_50_ from MTT
	(*μ*g/mL)	(*μ*g/mL)	(*μ*g/mL)	(*μ*g/mL)
PANC-1	3.19	1.67	56.46	26.81
hTERT-HPNE	8.06	3.74	160.3	58.46
	2.6 nm AgNPs		18 nm AgNPs	

The inhibitory concentration, IC_50_, was calculated from the following equation: log (inhibitor) versus responses curve using the GraphPad Prism 5 program.

## Data Availability

The data used to support the findings of this study are included within the article (Figures 1–7). Previously reported, our data (Table 1) were used to support this study and are available at (https://doi.org/10.18632/oncotarget.22563). These prior studies (and datasets) are cited at relevant places within the text as references [12].
